# South American Nothochrysinae (Neuroptera, Chrysopidae): I. Description of *Nothochrysaehrenbergi* sp. nov.

**DOI:** 10.3897/zookeys.866.35394

**Published:** 2019-07-24

**Authors:** Catherine A. Tauber

**Affiliations:** 1 Department of Entomology, Comstock Hall, Cornell University, Ithaca, NY 14853, USA Cornell University Ithaca United States of America; 2 Department of Entomology and Nematology, University of California, Davis, CA, 95616, USA University of California Davis United States of America

**Keywords:** *
Archaeochrysa
*, Chile, fossils, Green lacewing, wing venation

## Abstract

A new species, *Nothochrysaehrenbergi***sp. nov.**, is described from Chile; it is the first species of *Nothochrysa* to be reported from the Southern Hemisphere and only the second from the New World. The genus now contains six extant species as well as two species known from late Oligocene and Miocene fossils. An updated catalog of the valid *Nothochrysa* species is presented, and three *nomina dubia* are discussed. The inclusion of the new species in *Nothochrysa* is well supported by morphological features. However, it and other species currently in the genus also share significant features with *Archaeochrysa*, an older genus of Nothochrysinae which is known only from the Eocene (Ypresian) to the late Oligocene. It therefore appears that *N.ehrenbergi* is among the least derived *Nothochrysa* species, and that the separation of *Archaeochrysa* from *Nothochrysa* is open to question and further examination.

## Introduction

The family Chrysopidae currently consists of three extant subfamilies. Chrysopinae, with approximately 75% of the known chrysopid genera, is by far the largest (*N* = ~80 genera). The other two subfamilies combined are much smaller (*N* =14 genera): Apochrysinae with five genera (Winterton and Brooks 2002) and Nothochrysinaewith nine (Adams 1967, Adams and Penny 1992). In addition, Nothochrysinae has 13 genera known only from fossils (Makarkin and Archibald 2013, Archibald and Makarkin 2015). Based on its morphological characters and substantial presence in the fossil record, the subfamily Nothochrysinae has long been considered the most basal of the extant chrysopids. However, recent molecular evidence does not consistently support this conclusion (Engel et al. 2018, Winterton et al. 2019).

Currently, there are records of four extant genera of Nothochrysinae from the New World, three of which are endemic to the region: *Asthenochrysa* Adams & Penny and *Leptochrysa* Adams & Penny (one species each) in South America, and *Pimachrysa* Adams (five species) in North America. The fourth genus, *Nothochrysa* McLachlan, is widespread throughout the Northern Hemisphere, but only one species is known from the New World (western North America).

During the last few years, several very interesting specimens of Nothochrysinae from the New World were found in museums. Among these specimens is a new species of *Nothochrysa*, the first from South America and the first from the Southern Hemisphere. The article here describes this new species and discusses its possible relationships with other genera of Nothochrysinae. Also included among the recently discovered New World specimens is the second known example of *Leptochrysaprisca* Adams & Penny. A separate article redescribes and provides images of this rare monotypic genus (Tauber 2019).

### Systematics of *Nothochrysa* McLachlan

The genus *Nothochrysa* has had a tortuous taxonomic history that is well summarized by Tjeder (1966: 264). Briefly, over the years *Nothochrysa* has included a large number of species that correctly have been moved to other genera, mostly *Italochrysa* Principi. By the time this study began, the number of species in the genus *Nothochrysa* had been reduced to only ten – eight extant and two known from fossils (Oswald 2018). However, among the extant species there are three whose validity has been questioned. Thus, with the addition of the new species described here, there are eight confirmed, valid species of *Nothochrysa*: six extant and two from fossils (Table 1), as well as three *nomina dubia* (Appendix 1).

**Table 1.** Catalog of valid species names in the genus *Nothochrysa* McLachlan.

**Extant species**
*californica* Banks, 1892 [North America: southwestern Canada, western USA]
*capitata* (Fabricius, 1793) [Europe: widespread; northern Africa: Algeria, Tunisia]
*ehrenbergi* sp. nov. [South America: Chile]
*fulviceps* (Stephens, 1836) [Europe: widespread]
*sinica* Yang Chi-kun, 1986 [Asia: China]
*turcica* Kovanci & Canbulat, 2007 [Eurasia: Turkey]
**Fossil species**
*praeclara* Statz, 1936 [Miocene: Germany]
*stampieni* Nel & Séméria, 1986 [Oligocene: France]

## Material and methods

Current usage of terms for veins in neuropteran wings is largely based on the classic studies of tracheal pathways by Tillyard (1916) and Comstock (1918, and his earlier studies with Needham), which were later modified and interpreted by others, e.g., Adams (1967), Kukalová-Peck (1991), Kukalová-Peck and Lawrence (2004), and most recently Breitkreuz et al. (2017). I did not examine tracheal pathways in the current study, and this report uses terminology for veins and cells based on a combination of the above studies. For example, as is customary, the names of the primary veins are abbreviated and capitalized (e.g., C, costa; Sc, subcosta; R, radius; M, media; Cu, cubitus; A1, A2, A3, first, second, and third anal veins; also Psm, pseudomedia and Psc, pseudocubitus). When veins split, I use A and P to indicate the anterior and posterior branches, as proposed by Breitkreuz et al. (2017). In addition, the term “furcation” and its italicized abbreviation “*f*” are useful in referring to the point on a vein where it forks or splits. Thus, for example, M*f* applies to the point on the media where it splits into two branches, the media anterior, MA, and the media posterior, MP.

The names of crossveins are in lowercase, contain a hyphen, and often begin with a number; for example, 1c-sc is the first (basal-most) costal-subcostal crossvein. Cell names are written in lowercase, italicized, and often appended with a number; e.g., *csc1* refers to the basal-most cell between C and Sc. For historical and grammatical consistency, I retained the traditional prefix “intra”, rather than “inter” (as proposed by Breitkreuz et al. 2017), when referring to cells between two branches of the same major vein. For example, *im1* denotes the first “intramedian” cell, and *icu3* denotes the third “intracubital” cell. I also reversed the terms "eutriangular" and "pseudotriangular", as used by Breitkreuz et al. (2017) to categorize two types of *im1* cells. Their figure 17B, in which the *im1* is labeled "pseudotriangular", illustrates a triangular cell with three angles where three entities – two veins (MA, MP) and a crossvein (ma-mp) – intersect. This configuration is a true triangle and should carry the term "eutriangular". Similarly, their figure 17A illustrates another triangular-shaped *im1* cell, but this one has two curved sides (MA, MP) and only two angles where the veins intersect. They identified this configuration as "eutriangular", whereas it should be considered "pseudotriangular". The above changes do not affect the authors' interpretation of the venation, nor do they affect figures 17C or 17D. They merely help facilitate grammatical and user-friendly terminology.

The terminal traces of the various major veins were estimated by following the marginal branches basally to their origins on major veins (Fig. 2a, b). In some cases, it is not clear whether a pathway involves actual fusion and/or furcation of longitudinal veins versus the loss and/or insertion of a crossvein. In these cases, marginal veins can be traced to more than one basal origin. Thus, for consistency, the veins within the various areas indicated on Fig. 2a, b are those whose basal-most origin reasonably falls within the indicated field. Given the difficulty in deciphering the fusions and splitting of veins involved in the pseudomedia and pseudocubitus, it is understood that some veins at the margins of each field may stem from more than one basal vein. [Note: For both the forewing and hindwing, I assume that the CuA actually extends distally towards and meets the MP, as opposed to being connected to it via a crossvein. It would be of value to confirm this assumption, via tracheal examination of both wings.]

To avoid uncertainty, it is also worthwhile to mention the terms that refer to the orientation of the wing: anterior – toward the elongate margin on the upper (costal) edge of the wing; posterior – toward the elongate margin along the lower edge of the wing; basal or proximal – toward the inner edge of the wing attached to the body; apical or distal – toward the far, outer edge of the wing.

The terminology for other body parts follows common usage.

### Nothochrysa ehrenbergi

Taxon classificationAnimaliaNeuropteraChrysopidae

Tauber sp. nov.

4cffa2d7-debb-58a0-9186-e3da99dd4cc7

http://zoobank.org/528B2ED3-82DF-4A61-8DF2-DD9DD5D77FED

#### Type material.

The **holotype** (a male) is in the California Academy of Sciences (CAS). Its labels read: [1] “CHILE: Nuble [Ñuble] / Las Trancas / 20/25-II-1980 / Luis E. Pena [Peña]”; [2] “Suarius / flavescens / (Blanchard) / det. N. Penny, 1988”; [3] “HOLOTYPE / *Nothochrysa / ehrenbergi* / Tauber 2019” (Fig. 7f).

This single specimen was found in the CAS collection among the unidentified chrysopids. A subsequent search of the collection did not yield additional examples. Norm Penny’s ID label remains on the specimen but was not included in Fig. 7f. It refers to *Suariusflavescens*, a species that now is placed in Chrysopodes (Neosuarius), and with which the new species shares similar coloration and appearance (see Tauber 2010).

When discovered, the specimen was discolored, and its wings were loosely folded around its body. One pair of wings was removed for study and is now attached with water-soluble hide glue to a card mounted on the pin below the specimen. The other pair fell off and was reattached to the specimen with hide glue. The abdomen was cleared and dissected; it is preserved in glycerin within a genitalia vial attached to the pin.

#### Diagnosis.

**Figure 1.**
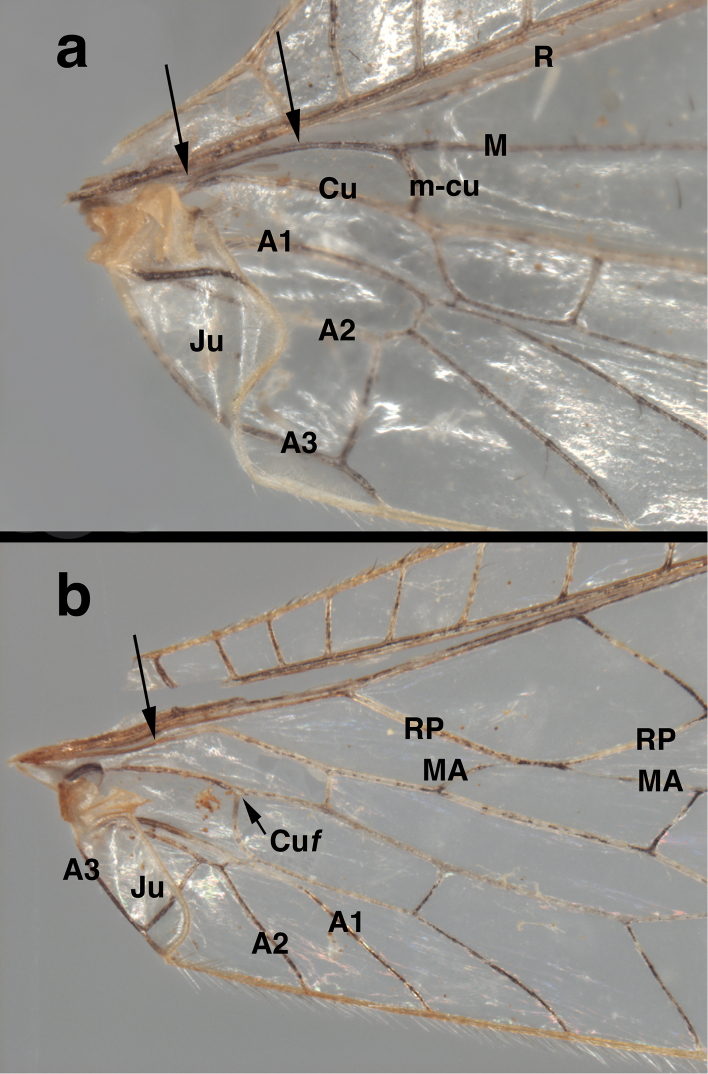
*Nothochrysaehrenbergi* sp. nov. (Ñuble, Chile; Male, CAS): Venation at base of wings (**a**) left forewing, (**b**) left hindwing. Note the absence of a tympanal organ at the base of R in the forewing, the independent origin and trajectory of M along the base of R (arrows pointing downward, both wings), and the alignment of RP and MA in the hindwing. **A1, A2, A3** first, second, third anal veins **Cu** cubitus **Cu*f*** furcation (division) of cubitus **Ju** jugal lobe **M** media **MA** media anterior **m-cu** media-cubital crossvein **R** radius **RP** radius posterior.

**Figure 2.**
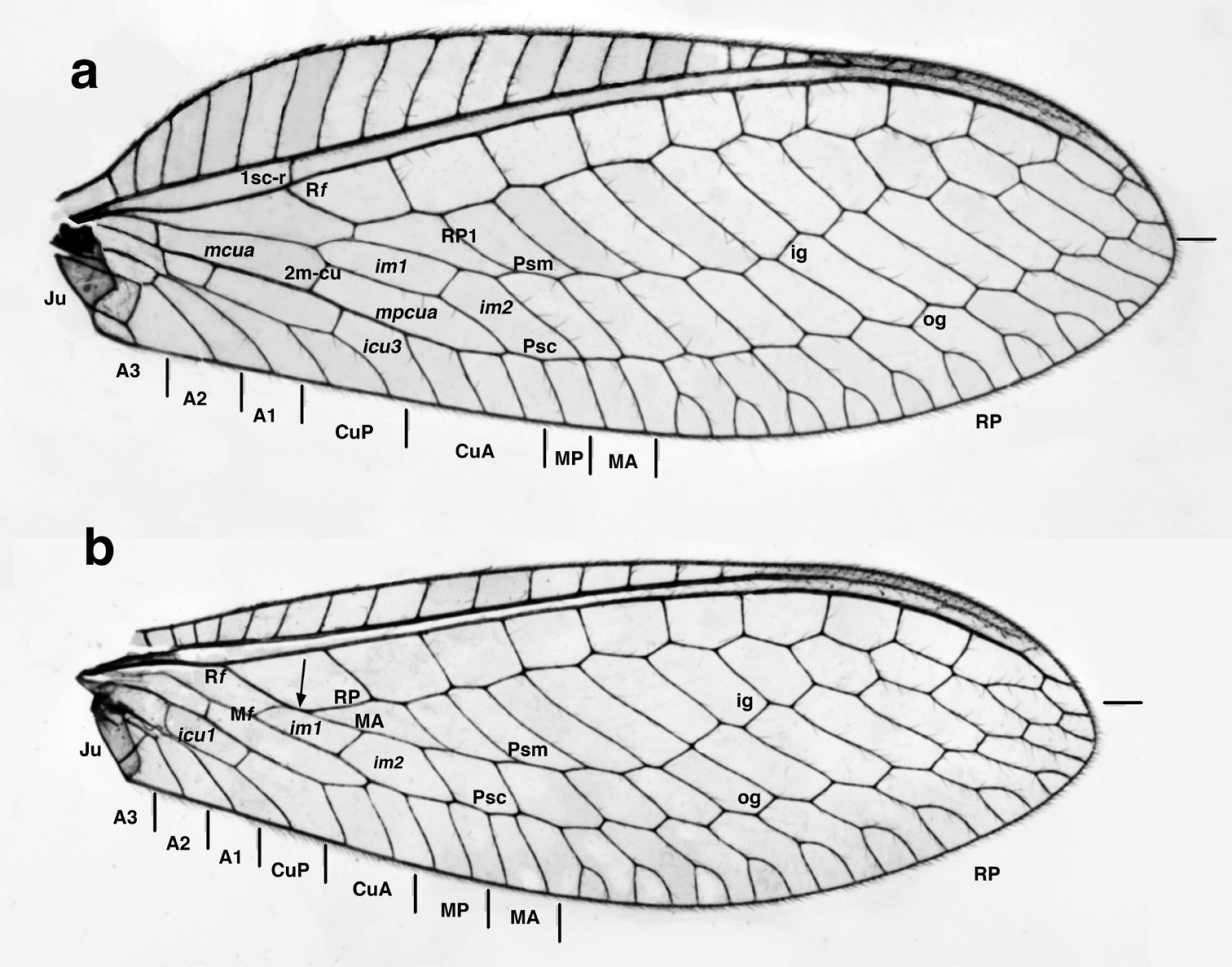
*Nothochrysaehrenbergi* sp. nov. (Ñuble, Chile; Male, CAS): Wings with selected features labeled (**a**) left forewing, (**b**) left hindwing. Marginal traces of major veins demarcated; arrow (hindwing) indicates alignment of RP and MA along upper margin of first intramedian cell. **A1, A2, A3** first, second, third anal veins **CuA, CuP** anterior, posterior branches of cubitus ***icu1*, *icu3*** first, third intracubital cells **ig** inner gradate ***im1, im2*** first, second intramedian cells **Ju** jugal lobe **MA** media anterior **MP** media posterior ***mcua, mpcua*** second and third medial cells **M*f*** furcation of media **og** outer gradate **Psc** pseudocubitus **Psm** pseudomedia **R*f*** furcation of radius **RP** radius posterior **RP1** first branch of radius posterior **1sc-r** first crossvein between subcosta and radius **2m-cu** second crossvein between media and cubitus.

**Figure 3.**
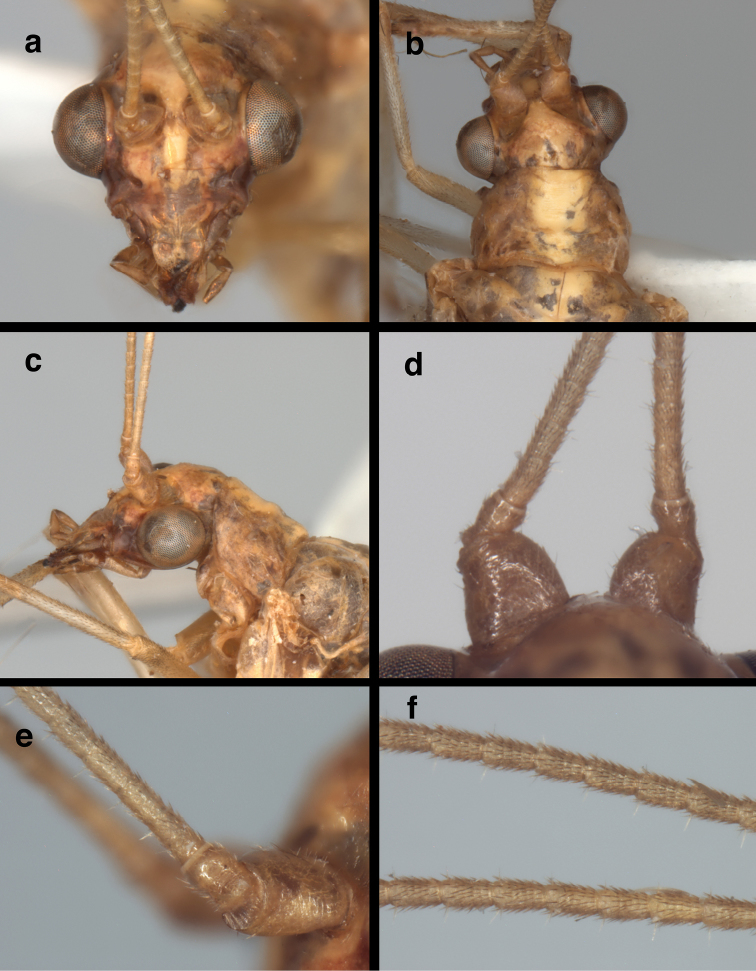
*Nothochrysaehrenbergi* sp. nov. (Ñuble, Chile; Male, CAS): Head and prothorax (**a**) head, frontal (**b**) head and prothorax, dorsal (**c**) head and prothorax, lateral (**d, e**) base of antennae, dorsal, lateral **(f)** flagellar segments, mid antenna.

**Figure 4.**
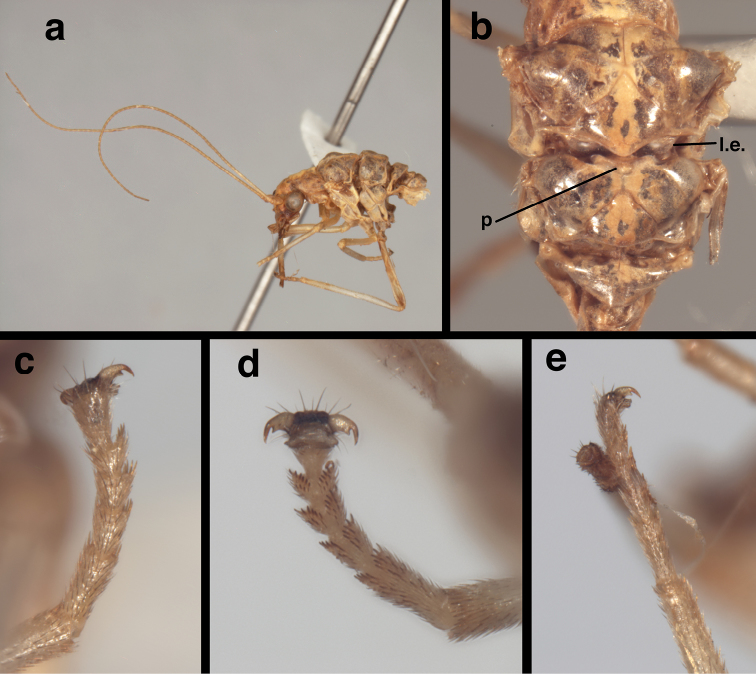
*Nothochrysaehrenbergi* sp. nov. (Ñuble, Chile; Male, CAS): Habitus (**a**) antenna, head, and thorax, lateral (**b**) mesothorax, metathorax, dorsal (**c**) metatarsus, dorsal **(d)** metatarsus, ventral **(e)** mesotarsus, lateral. **p** raised metascutal protuberance **l.e.** mesoscutellar lobate expansion.

Subfamily: This specimen exhibits the following diagnostic features of adult Nothochrysinae (cf.: Tjeder 1966, as Dictyochrysinae; Adams 1967; Brooks and Barnard 1990; Makarkin and Archibald 2013; Breitkreuz 2018): (i) wing-coupling mechanism consisting of a large jugal lobe on the forewing (here, folded ventrally; Fig. 1) and a frenulum on the hindwing (here, broken off); (ii) base of the forewing without tympanal organ (Fig. 1); (iii) forewing (and hindwing) with stem of the media extending basally, adjacent to the radius and not fused with it (Fig. 1a, b; cf. Breitkreuz et al. 2017: 32); (iv) first intramedian cell triangular, with boundaries formed by the MA, the MP, and the crossvein 1ma-mp ("pseudotriangular", sensu Breitkreuz et al. 2017); (v) pseudomedia ill-defined or appearing to merge with inner (not outer) series of gradates (Fig. 2); (vi) pseudocubitus appearing to merge with outer series of gradates (Fig. 2); (vii) forewing with basal subcostal crossvein present (Fig. 2); (viii) second m-cu crossvein stemming from the proximal half of the first intramedian cell (Fig. 2); (ix) each flagellomere having five or six whorls of setae (Figs 3e, 3f); and (x) anterodorsal surface of the metascutum displaying small, convex protrusion (Fig. 4b; cf. Breitkreuz 2018, Tauber 2019).

Genus placement: The Chilean specimen under study here falls into the genus *Nothochrysa* on the basis of the following features of its wings (Figs 1, 2): (i) forewing and hindwing having well developed pseudomedia and pseudocubitus; (ii) forewing and hindwing with two regular series of gradate veins (inner and outer); (iii) intramedian cell of forewing triangular, elongate, occupying approximately half the width between the pseudomedia and pseudocubitus; (iv) RP of forewing with 10 or more branches (Adams 1967; Makarkin and Archibald 2013; Archibald and Makarkin 2015; Breitkreuz 2018: 200). [Note: Some specimens of *N.californica* are known to have only eight or nine branches from the RP.]

Species placement: Apart from being the only known *Nothochrysa* species reported from South America, *N.ehrenbergi* is distinguishable from other species of *Nothochrysa* on the basis of a number of wing characters (Figs 1, 2; cf. Adams 1967; Aspöck et al. 1980: figs 154, 155; Kovanci and Canbulat 2007: fig. 2): (i) the first anal vein is not forked; (ii) the basal subcostal crossvein is slightly distal to the furcation of the radius; (iii) as in most *Nothochrysa* species, the first intramedian cell is more wedge shaped than truly quadrangular or triangular (i.e., the MA and MP meet basally at a broadly acute angle); and (iv) the third medial cell (directly below *im1*, Fig. 2a) is elongate and extends toward the pseudocubitus well beyond the distal edge of first intramedial cell.

#### Morphological characteristics.

Head (Fig. 3): Width 1.6 mm (including eyes); ratio of head width to eye width = 3.0 : 1. Vertex raised, round; surface pitted anteriorly, with few or no setae, lacking prominent posterior fold. Distance between scapes 0.09 mm; distance between tentorial pits 0.36 mm; length of frons (midway between scapes – midway between tentorial pits) 0.33 mm. Frons relatively wide, with broad longitudinal ridge mesally; surface smooth, shiny, slightly rounded below toruli and at insertion of mouthparts; margin above clypeus straight. Clypeus tapering, with rounded sculpturing basally, indented mesally, slightly expanded distally, with distal margin straight to slightly convex; dorsal surface shiny, smooth, sculptured. Labrum about same width as clypeal margin, with small longitudinal ridge mesally; dorsal surface sculptured, shiny; distal margin bilobed, bearing numerous long setae distally. Antenna 9.7–9.8 mm long (~0.5× length of forewing); scape shorter than wide (0.23 mm long, 0.33 mm wide), lateral margin straight, mesal margin strongly convex, surface with short setae throughout; pedicel 0.17 mm long, 0.13 mm wide, with numerous short setae; flagellum with basal flagellomeres distinct, somewhat elongate (0.12–0.14 mm long, 0.07–0.08 mm wide), midantennal flagellomeres twice as long as broad (0.15 mm long, 0.07 mm wide), basal two flagellomeres with 4–5 partially indistinct whorls of thickset brown setae extending distally, third flagellomere and others distally all with five distinct whorls of thickset, brown setae extending distally, 0.3–0.5× width of flagellomere, distal whorl with one or two slender, elongate (~0.75× width of flagellomere), pale setae extending laterally.

*Head coloration*: Scape cream, with reddish spot on distolateral tip; pedicel, flagellum cream, unmarked; thickset setae in whorls mostly brown, elongate setae pale. Vertex cream, possibly tinged red laterally; dorsal torulus yellow to cream, apparently unmarked. Frons cream, probably with reddish tinge laterally below torulus; torulus cream, unmarked. Clypeus cream, possibly tinged red laterally; basal, distal margins straight. Genal mark dark red/brown throughout, extending to tentorial pit. Labrum probably cream. Palpomeres probably mostly cream, somewhat darkened distally.

Thorax (Fig. 4): Cervix not visible. Dorsal thoracic surface with pale longitudinal stripe mesally, probably with broad reddish or brownish stripes or coloration laterally. Prothorax broad, 0.9 mm long, 1.5 mm wide, ratio of length to width = 0.63 : 1; pronotum well sclerotized, with textured surface, transverse fold mesally, few or no setae. Legs elongate, slender, probably cream, unmarked, lacking prominent tibial spurs. Tarsus with basal three tarsomeres appearing coalesced, bearing spurs, setae intermixed along undersurface; middle three tarsomeres with expanded lateral lobes bearing spurs, setae in irregular rows; distal tarsomere narrow basally, enlarged distally, bearing numerous elongate, slender, dark setae laterally, distally, terminus bearing pair of claws laterally, large pad mesally; claw amber, with basal enlargement, acute slender hook terminally.

**Figure 5.**
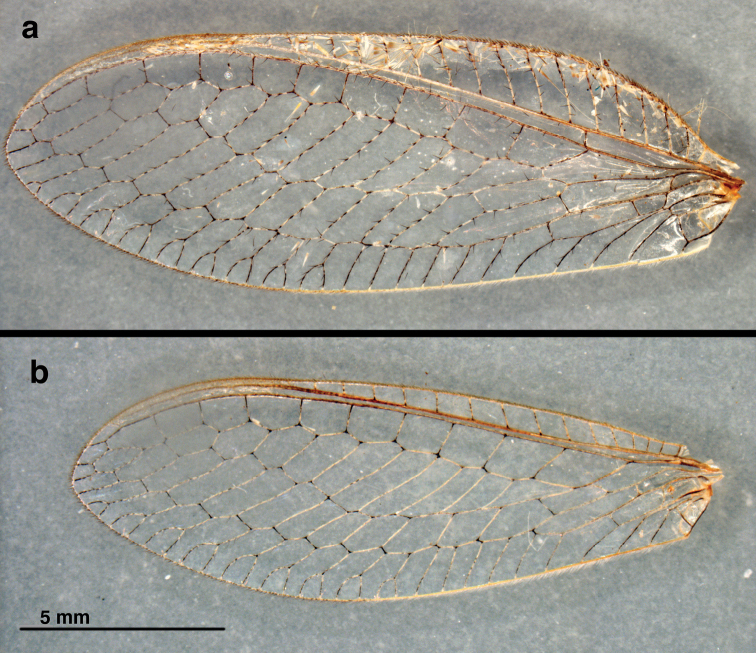
*Nothochrysaehrenbergi* sp. nov. (Ñuble, Chile; Male, CAS): Wings, color slightly enhanced to emphasize pattern of vein markings (**a**) forewing (**b**) hindwing.

Wings (Figs 1, 2, 5): *Forewing* 18.5 mm long, 6.5 mm wide (at widest point); ratio of length to maximum width = 2.9 : 1. Membrane clear, lacking markings; microtrichia present below base of every major vein, pale. Trichosors (sensu Makarkin and Archibald 2013: 140–142) absent. Costal area relatively enlarged; tallest costal cell (7^th^ from base of wing) 1.8 mm tall, 2.7× width of cell, 0.28× height of wing; costal crossveins simple, six before 1sc-r, twelve after 1sc-r and before stigma, one (very small) after stigma, none within stigma. Sc extending into stigma, fading but not appearing to merge with C or RA; no crossveins in stigma; first sc-r crossvein slightly distal to R*f*, slightly basal to M*f*; RA with one very short veinlet extending to wing margin after stigma. Radial area between RA and RP with single row of ten closed cells; tallest cell (3^rd^ from base of wing) 0.6× as tall as wide. Intramedian cell (*im1* = *mamp1*) prominent, elongate, triangular, formed by MA, crossvein 1ma-mp, and two abscissae of MP, occupying approximately half the space between MA and CuA, with M*f* broadly acute, long sides (MA, MP) roughly parallel for most of span; crossvein 2m-cu proximal to midpoint of *im1*. Three medial cells present (*mcu, mcua, mpcua*), second, third of these elongate, with roughly parallel sides; MP merging into Psc well beyond *im1*. Two series of gradate veins parallel basally, diverging slightly medially, converging distally. Approximately nine inner gradates in regular, sinuous series, continuing from Psm in zigzag pattern across center of wing; approximately ten outer gradates continuing from Psc in regular, upturned series. RP with nine marginal forks beyond Psc. Cu furcated after m-cu crossvein, with two closed, four open *icu* cells. CuA with three furcations before meeting MP; CuP furcated below *icu2*; thus cubital trace having five terminal veinlets (three from CuA, two from CuP). A1, A2, A3 simple, unforked; a1-a2 and a2-a3 crossveins present; distal part of *a3* and jugal lobe with dense patch of microtrichia. Jugal lobe large, quadrate, folded beneath third anal cell, without internal vein; margin bearing long, slender setae basally.

*Hindwing*: 12.4 mm long, 4.2 mm wide. Costal area not enlarged; at least 15 c-sc crossveins before stigma, none within or after stigma. Radial area containing single row of eleven closed cells between RA and RP. Gradate veins in two roughly parallel series, slightly divergent distally; approximately seven inner gradates beyond Psm; approximately 11 outer gradates beyond Psc. Psc with nine marginal forks. MA aligned with RP for approximately one-third length of *im1*. CuA with two furcations before meeting MP; CuP undivided; thus, wing margin having three cubital veinlets (two from CuA, one from CuP). A1, A2, A3 simple, unforked; a1-a2 and a2-a3 crossveins present. Jugal lobe without internal vein, basal margin bearing long, slender setae.

*Coloration of forewing, hindwing* (Fig. 5): Membrane clear, somewhat glossy. Stigma slightly opaque, without coloration. Costal, subcostal, radial veins brownish; all other longitudinal veins pale with black marks at intersections and (forewing) at bases of setae. Forewing with posterior veinlets extensively marked black; basal inner gradates pale, others becoming increasingly marked black until entirely black distally; outer gradates mostly black. Hindwing with basal inner gradates pale, marked with black at intersections; outer gradates mostly black.

**Figure 6.**
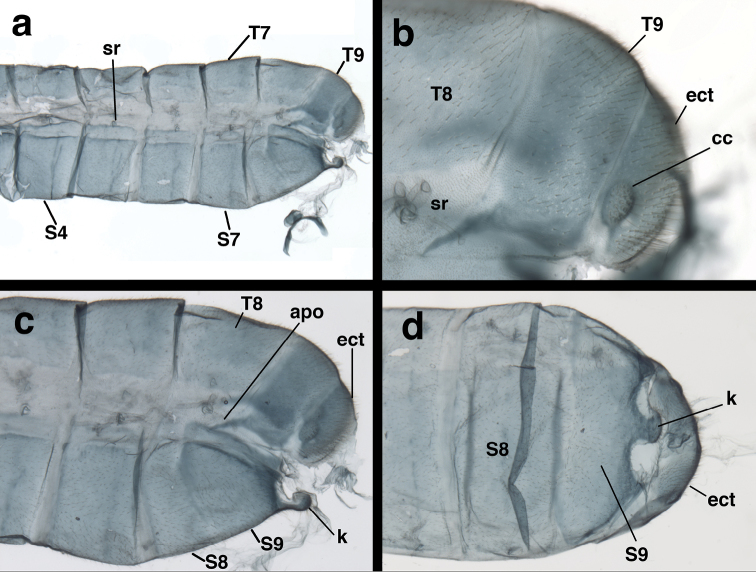
*Nothochrysaehrenbergi* sp. nov. (Ñuble, Chile; Male, CAS): Abdomen, cleared (**a**) midsection-terminus, lateral (**b**) T8 (distal), T9, and ectoproct, lateral (**c**) terminal abdominal segments, lateral (**d**) terminal abdominal segments, ventral. **apo** dorsal apodeme extending below T8 **cc** callus cerci **ect** ectoproct **k** distal knob extending from S8+9 **sr** spiracle **S4, S7** fourth, seventh strenites **S8, S9** partially coalesced eighth and ninth sternites **T7, T8, T9** seventh, eighth, ninth tergites.

Abdomen (Male, Fig. 6; female unknown): Sclerites, integument of pleural region somewhat soft, flexible; tergites, sternites, pleural region covered with setae of uniformly short length; microsetae present, no microtholi. T6: length 0.78 mm, ~1.8× height; T7: length 0.80 mm, ~1.6× height; S6: length 0.67 mm, 0.72× height; S7: length 0.68 mm, ~0.70× height. Tergites roughly rectangular, edges acute or slightly rounded, ventral margins straight or slightly concave mesally. Spiracles located approximately in center of lateral membrane, roughly circular externally, not enlarged; atria slightly enlarged, rounded, with bifurcated tracheae. Coloration: body somewhat discolored; setae pale. Tergites probably green, without markings; pleuron mostly tan; sternites with green longitudinal stripe dorsally, tan ventrally; callus cerci white.

**Figure 7.**
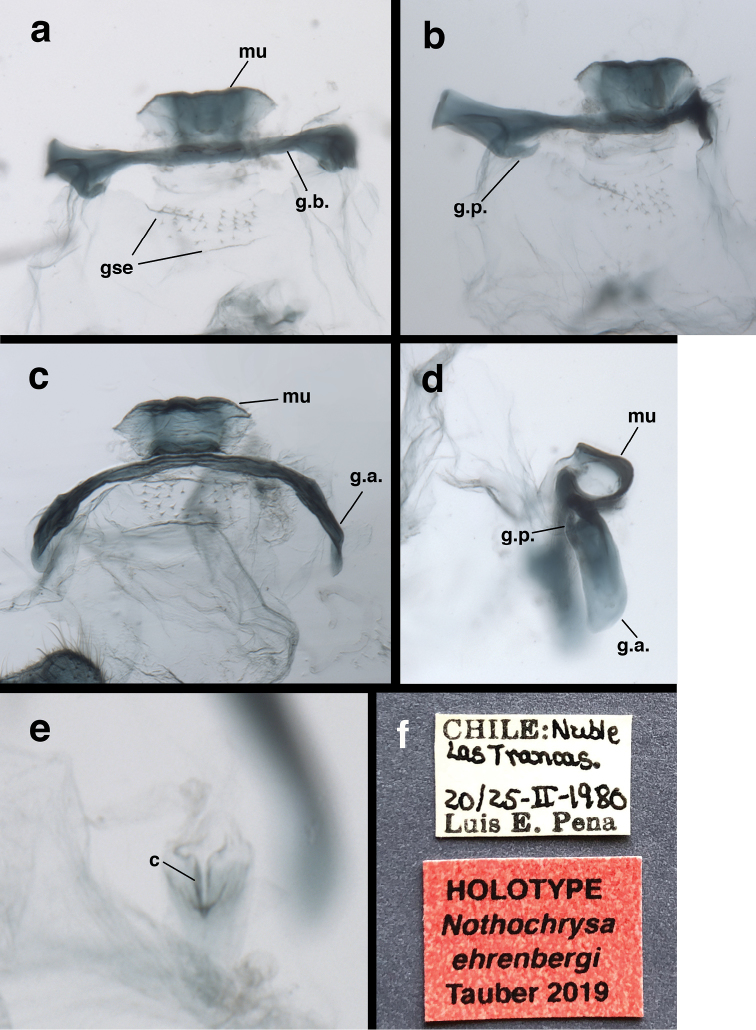
*Nothochrysaehrenbergi* sp. nov. (Ñuble, Chile; Male, CAS): Male genitalia, cleared, and specimen labels (Penny’s identification label not included) (**a**) gonarcal complex, dorsal (**b**) gonarcal complex, frontal, tilted (**c**) gonarcal complex, posterior (**d**) gonarcal complex, lateral (**e**) hypandrium internum **(f)** labels. **c** comes **g.a.** gonarcal apodeme **g.b.** gonarcal bridge **g.p.** gonarcal process **gse** gonosetae on membranous gonosaccus **mu** mediuncus.

Male terminalia (Fig. 7): T8 broadly wedge shaped, with dorsal surface slightly rounded, length 0.83 mm, height 0.49 mm, considerably longer than dorsal surfaces of either T9 or ectoproct; lateral margins tapering inward ventrally, ventral margin roughly straight. T9 and ectoproct separate, not fused; callus cerci ovate, protruding basally from posterior margin of ectoproct, 0.18 mm length, 0.10 mm width, with ~30 trichobothria of various lengths. T9 rectangular, with distoventral margin rounded; elongate, lightly sclerotized ventral apodeme along ventral margin, extending proximally to midsection of A8. Ectoproct dome shaped, rounded distally, slightly convex basally, tightly curved ventrally, sloping dorsally; callus cerci situated on lower proximal margin. S8 and S9 partially fused, without internal ridge; S9 more heavily sclerotized than S8, posterior margin slightly more sclerotized than remainder of sternite. S8+9 (lateral view) with proximal margin straight ventrally, becoming broadly rounded dorsally, distal margin short, straight, ventral margin straight; terminal knob extending well beyond edge of S9, with elongate setae on ventral margin; dorsal surface of knob contiguous with heavy recurrent membrane attached to elongate gonarcal membrane. Subanal plate not found.

Gonarcus delicate, slender, broadly arcuate; lateral apodemes slender, quadrate (lateral view), rounded distally, with short, contiguous processes mesally, extending forward. Mediuncus closely attached to dorsal surface of gonarcal arch, flat, recurved into an almost fully circular hood, with two internal sclerotized “rods” extending roughly in parallel from mediuncal base to tip, converging slightly at tip; base of mediuncus quadrate (dorsal view), occupying approximately one-fourth span of gonarcal bridge; terminus of mediuncus with expanded lateral wings, rounded mesal protrusion. Gonosaccus transparent, immediately beneath gonarcal arch and mediuncus, with approximately 32 short setae on distinct setal bases uniformly distributed in two equal patches. Hypandrium internum small, located on delicate membrane extending well below gonosaccus, consisting of paired, curved lateral arms meeting mesally at narrow, rounded apex; comes lightly sclerotized, extending forward beyond apex. Gonapsis, gonocristae absent.

#### Biology.

Nothing is known about the biology or larval morphology of this species. The gut of the *N.ehrenbergi* specimen did not contain noteworthy contents.

Larval descriptions of several *Nothochrysa* species are available for comparison if *N.ehrenbergi* larval specimens were to become available (see Tauber et al. 2014). *Nothochrysa* larvae generally are considered debris-carriers, but their packets of debris are small, and their morphology is only moderately modified for debris-carrying. In addition, detailed information on aspects of the developmental and reproductive biology of *N.californica* is available (Toschi 1965).

For generic-level comparisons, larval descriptions for genera within Nothochrysinae (*Kimochrysa*, *Pimachrysa*, *Dictyochrysa*, and *Hypochrysa*) have been published (see Tauber et al. 2014). Unfortunately, larvae of *Asthenochrysa, Leptochrysa, Pamochrysa*, and *Triplochrysa* are not described.

#### Known distribution.

Currently, this species has only been reported from the type locality, which presumably is the Valle Las Trancas in the region of Ñuble, Chile.

#### Etymology.

This species is named in honor of Ronald G. Ehrenberg, Irving M. Ives Professor of Industrial and Labor Relations and Economics at Cornell University, an esteemed and cherished colleague of the author and her late husband (Maurice J. Tauber).

##### Characteristics shared with *Archaeochrysa* species

As shown above, *N.ehrenbergi* shares many features with other extant *Nothochrysa* species, and its inclusion in the genus is well supported. However, the species also expresses many features that differ from *Nothochrysa* and that are shared by at least some of the five species in the fossil genus *Archaeochrysa*. I discuss four below:

First, in the *N.ehrenbergi* forewing, vein A1 is not forked, whereas it is forked in all other *Nothochrysa* species (Adams 1967; Aspöck et al. 1980: figs 154, 155; Makarkin and Archibald 2013: 135, 136). The feature is variable in *Archaeochrysa* specimens where A1 is visible. It is not forked in two species (Adams 1967: 237), forked in two species (Adams 1967: 230, Makarkin and Archibald 2013: 135), and missing from the specimen of the fifth species (Archibald and Makarkin 2015: 363).

Second, in *N.ehrenbergi* the basal sc-r crossvein arises distal to the furcation of the radius and almost directly above the furcation of the media. Both of these character states are shared with the fossil genus *Archaeochrysa* (Adams 1967, Makarkin and Archibald 2013), but not with other known *Nothochrysa* species.

Third, in *N.ehrenbergi* the distinction between the inner gradate series and the pseudomedia as well as between the outer gradate series and the pseudocubitus is indistinct. Rather, the gradate series and their respective pseudoveins tend to run together more smoothly as a curve, rather than at an angle as in other *Nothochrysa* species. Again, this feature of *N.ehrenbergi* is shared most closely with *Archaeochrysa* species (Adams 1967, Makarkin and Archibald 2013, Archibald and Makarkin 2015).

Fourth, currently the primary feature used to distinguish between *Nothochrysa* and *Archaeochrysa* is the presence or absence of a crossvein between RP and MA in the basal part of the hindwing. The crossvein is present in all known *Archaeochrysa* species and is reported to be absent from *Nothochrysa* (Makarkin and Archibald 2013: 134). In *N.ehrenbergi*, MA aligns with RP for about one-third the length of the upper margin of the *im1* cell, and no crossvein is present (Figs 1b, 2b). However, even with this character there appears to be a possible exception. Figure 2 accompanying the original description of *Nothochrysaturcica* Kovanci and Canbulat shows a short crossvein between RP and MA; confirmation of the accuracy of this drawing is necessary.

##### Phylogenetic position of *Nothochrysaehrenbergi* sp. nov.

Given the above, Archibald and Makarkin’s (2015) discussion of the phylogeny of *Archaeochrysa* species is worthy of consideration here. Their paper evaluates how the various *Archaeochrysa* species express three features; each feature has several conditions ranging from presumably plesiomorphic to more derived. Below, the three features are considered, relative to their expression by *Nothochrysa* species, especially *N.ehrenbergi*.

(1) **The shape of the *im1* cell.**Archibald and Makarkin (2015) describe two configurations for this character; *N.ehrenbergi* expresses the second (more advanced) condition in which the sides of the *im1* cell are almost parallel for most of their span and converge basally at a relatively steep angle. The extant species of *Nothochrysa*, including *N.ehrenbergi*, share this feature with two species of *Archaeochrysa*.

(2) **The position of crossvein 2m-cu.**Archibald and Makarkin (2015) list six conditions for this character, each one considered more evolutionarily advanced than the preceding. *Nothochrysaehrenbergi* falls into Condition 5, a derived condition in which 2m-cu is located distinctly in the proximal part of *im1* (as shown in fig. 2C of Archibald and Makarkin 2015). This character state is typical of at least two *Archaeochrysa* species, *A.creedi* (Adams) and *A.paranervis* (Adams), as well as several other extant genera in Nothochrysinae, including *Nothochrysa*.

(3) **The crossveins of Psc.**Archibald and Makarkin (2015: 366) describe and illustrate four character states for this feature; interested readers are referred to the original paper. Suffice it to say here, *N.ehrenbergi*, as well as three *Archaeochrysa* species but no other *Nothochrysa* species, fall into the second of the four conditions. This position is considered plesiomorphic among Nothochrysinae, both fossil and extant (Archibald and Makarkin 2015).

On the basis of the above information, it appears that *N.ehrenbergi* shares a very close phylogenetic relationship with the fossil genus *Archaeochrysa*. At this point, only one character (the absence of a crossvein between the RP and the MA above the first intramedial cell of the hindwing) supports its exclusion from *Archaeochrysa*, and this character may have exceptions within *Nothochrysa*. Indeed, there does not appear to be a synapomorphic character that consistently differentiates *Nothochrysa* from *Archaeochrysa*. Thus, given the overall similarity between *N.ehrenbergi* and the known *Archaeochrysa* species, I recommend that future studies examine the validity of maintaining the generic separation.

## Supplementary Material

XML Treatment for Nothochrysa ehrenbergi

## Appendix 1. *Nomina dubia* within *Nothochrysa*

### *Nothochrysaindigena* Needham, 1909

Although Ghosh (1990: 349) may be correct in his inclusion of this species in Nothochrysinae and *Nothochrysa*, his evidence as published remains questionable. The main characteristic that argues in favor of the identification is his report of a tympanum being absent. However, several other features contradict the identification and lead me to question whether the tympanum was overlooked. First, his report mentions flagellomeres with four whorls of setae (not five or six whorls as is typical of *Nothochrysa* and Nothochrysinae in general). Second, the images of the forewing and hindwing show no jugal lobe, no frenulum, and no basal subcostal crossvein; nor is there any mention of the presence of these structures – all of which are features of Nothochrysinae, including *Nothochrysa*. Third, the shape of the *im1* cell is not elongate, and the MA and MP that form the upper and lower margins of the cell are not parallel as described for other species of *Nothochrysa*. Fourth, Ghosh reports that the pseudomedia merges with the inner gradates; however, his figures illustrate the Psm intersecting (not merging) with the inner gradates well before the end of the Psm and at a much steeper angle than in any known *Nothochrysa* species (Ghosh 1990: figs 16, 17). While the configuration of the Psm in *Nothochrysacalifornica* Banks is similar to that of Chrysopinae, it does not resemble that which is depicted in the figure of *N.indigena*. Fifth and finally, the spinose tip of the male S8+9 (Ghosh 1990: fig. 19) is unusual for *Nothochrysa*, and the systematic importance of this structure is unknown.

### *Nothochrysalefroyi* Needham, 1909

No published information is available that helps identify the generic placement of this species, and apparently the type specimen has not been found (Needham 1909: 203, Ghosh 1990: 351).

### *Nothochrysapolemia* Navás, 1917

The original description of this species is relatively detailed for its time, and it includes two illustrations. The type is reported from Mytilene, a city on the island of Lesbos in the North Aegean region (Navás 1917). Originally, the type was in Navás’ collection; however, it is not there now (Monserrat 1985), and it is believed to have been destroyed. The name was treated as a *nomen dubium* by Aspöck et al. (2001: 314), who considered the species likely to be synonymous with *Rexaraddai* (Hölzel 1966). The designation of a neotype is necessary (Oswald 2018).
